# Biomechanical characteristics of a novel interspinous distraction fusion device in the treatment of lumbar degenerative diseases: a finite element analysis

**DOI:** 10.1186/s12891-023-07066-6

**Published:** 2023-12-06

**Authors:** Mengmeng Chen, Jiechao Deng, Li Bao, Pu Jia, Fei Feng, Guan Shi, Hai Tang, Hao Chen

**Affiliations:** Department of Orthopaedics, Beijing Friendship Hospital, Capital Medical University, No. 95, Yong An Road, XiCheng District, Beijing, 100050 People’s Republic of China

**Keywords:** Interspinous distraction fusion device, Biomechanical properties, Lumbar degenerative diseases, Finite element analysis

## Abstract

**Background:**

A novel interspinous distraction fusion (ISDF) device has been used to treat lumbar degenerative diseases. As a minimally invasive technique, ISDF differs from the traditional interspinous process distraction devices. Currently, biomechanical studies on ISDF are rare.

**Objective:**

To investigate the biomechanical properties of the ISDF device (BacFuse) which is used to treat lumbar degenerative diseases.

**Methods:**

Three-dimensional L3-L5 models were created. The models were divided into four groups: intact (M1), local decompression alone (M2), internal fixation alone (M3) and local decompression combined with internal fixation (M4), based on different surgical procedures. Local laminectomy was performed to resect the lower part of the L4 lamina and the upper part of the L5 lamina at the right lamina of L4/5 in the M2 and M4 groups. After meshing the models elements, Abaqus were used to perform the finite element (FE) analysis. The intervertebral range of motion (ROM) was measured during flexion, extension, left lateral bending, right lateral bending, left rotation and right rotation under a follower load of 400 N with a 7.5Nm moment. The distributions of disc and facet joint stresses were observed and recorded. Spinal vertebral stress was compared, and internal fixation device stress was observed.

**Results:**

The ROM of L4/5 in M2 increased in flexion, extension, left lateral bending, right lateral bending, left rotation and right rotation compared with that in M1. In all motion directions, the ROM at L4/5 decreased, and the ROM at L3/4 increased after implantation of the ISDF device in M3 and M4 groups. The disc stress and facet joint stresses in the instrumented segment decreased after implantation of the ISDF device. The spinous process loaded a certain amount of stress in M3 and M4 groups. The spikes of the internal fixation device were loaded with the maximum stress.

**Conclusion:**

BacFuse exhibited a reduction in intervertebral ROM, as well as decreased stress on the intervertebral disc and facet joint, while also demonstrating a discernible impact on the upper adjacent segment.

## Introduction

Interspinous process distraction (IPD) is a minimally invasive technique that used in the treatment of lumbar spinal stenosis (LSS) [1]. The interspinous spacer as acts shock-absorber, resulting in indirect decompression [2]. The use of IPD devices has previously been controversial owing to high recurrence and revision rates during long-term follow-up [3, 4]. However, in recent years IPD devices has been improved through developments in medical science and technology [5]. Several novel IPD devices have been developed and applied in clinical settings. Moreover, indications for these devices are not limited to the treatment of LSS but extend to all types of lumbar degenerative diseases [6, 7].

BacFuse is a novel IPD device used to treat lumbar degenerative diseases [8]. It not only distracts the interspinous space for indirect decompression, but also firmly anchors it to the spinous process, preventing the potential instability caused by local laminectomy. Moreover, interspinous bone grafts allow interspinous fusion, thereby reducing the risk of internal fixation failure. Therefore, this type of IPD device is called interspinous distraction fusion (ISDF) device [9]. Currently, the ISDF devices are widely used in the clinic and have been reported to have good clinical efficacy. Raikar et al. reported that 13 patients with severe lower back pain and lumbar radiculopathy treated with ISDF showed a significant improvement in pain scores at a median follow-up of 19 months [10]. Falowski et al. reported on 32 cases of lumbar degenerative disc disease being treated with ISDF. The results indicate that ISDF is a valuable technique for the treatment of moderate to severe LSS with few complications and significant efficacy [11]. In our previous study, we found that ISDF is a viable method for octogenarian patients with LSS compared with traditional fusion surgery [9].

However, few biomechanical studies have been conducted on ISDF devices used in lumbar degenerative diseases. Liu et al. reported that three IPD devices (X-Stop, Coflex, and BacFuse) effectively reduced extension, and disc and facet joint stress, using a finite element (FE) analysis [12]. In fact, ISDF is often combined with unilateral local laminectomy. In the study by Spallone, 29 patients underwent BacFuse implantation as an adjunct to decompressive surgery, while 12 patients underwent BacFuse implantation as a stand-alone technique [8]. In Liu’s study, the biomechanical characteristics of BacFuse as an adjunct device following decompressive surgery were not analyzed. Therefore, we performed FE analysis to further explore the biomechanical characteristics of ISDF devices (BacFuse) in practical applications.

## Methods

### FE modelling of the lumbar spine

**Fig. 1:**
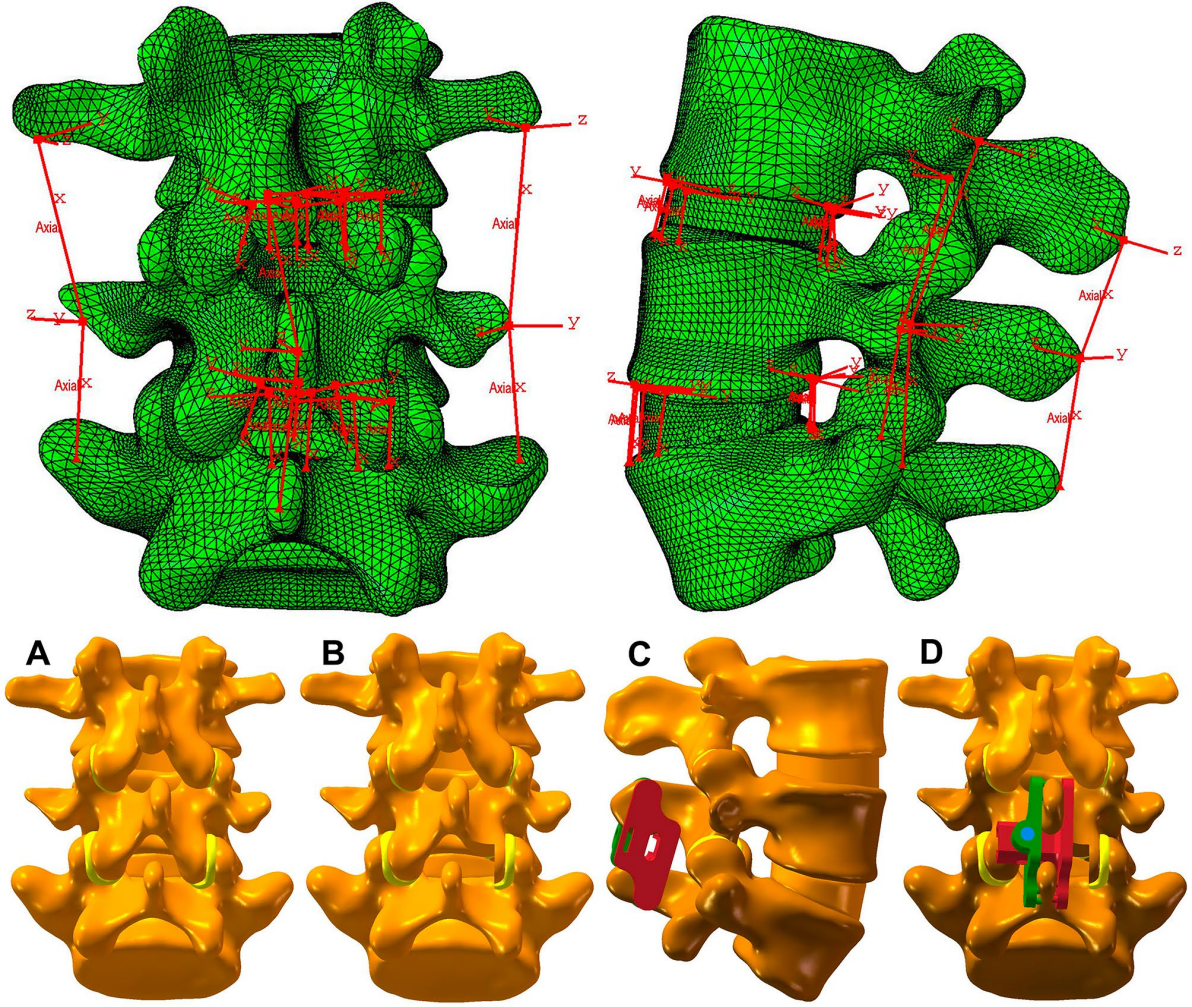
(**a**) The finite analysis model included three vertebrae (L3, L4 and L5), two IVDs (L3/4 and L4/5), four facet joints, and seven ligaments. (**b**) Four kinds of 3D lumbar models (L3-L5). (**A**) Intact model. (**B**) Unilateral decompression model. (**C**) Internal fixation alone model. (**D**) Unilateral decompression combined with internal fixation model

A lumbar disc herniation patient (52-year-old, male, BMI 23.56 kg/m^2^) was recruited to complete a lumbar spine CT (Discovery CT 750 HD, General Electric Company, Milwaukee, WI, USA) scan after signing an informed consent form. The CT data (slice thickness, 0.675 mm) in the DICOM format were stored in Mimics software version 11.0 (Materialise NV, Leuven, Belgium) and converted to a three-dimensional (3D) model. The 3D model was stored into Unigraphics NX (UG) software (Siemens Digital Industries Software, Plano, TX, USA) to construct the intervertebral disc (IVD) as a 3D digital model. The FE model was constructed after Hypermesh processing(Altair Engineering, Troy, MI, USA) to generate the mesh. The material property assignment and contact definition were completed using Abaqus software (Simulia, Johnston, RI, USA) (Table 1) [13, 14]. The FE model included three vertebrae (L3, L4 and L5), two IVDs (L3/4 and L4/5), four facet joints, and seven ligaments (anterior longitudinal, posterior longitudinal, ligamentum flavum, supraspinous, interspinous, intertransverse process, and capsular) (Fig. 1a). The outer 1 mm of every vertebra is cortical bone, and the inner part is cancellous bone [15]. The disc was divided into a nucleus pulposus and a fibrous annulus, with a volume ratio of 56 − 44%. The articular surface was simulated as a 0.5-mm thick cartilage layer, with a frictionless surface contact simulating a joint gap of < 1 mm. The seven ligaments were simulated as non-linear elastic materials under pure tension.

**Table 1:** Material properties

Component	Young’s modulus(Mpa)	Poisson ratio
Cortical bone	12,000	0.3
Cancellous bone	100	0.2
Endplate	24	0.4
Articular cartilage	25	0.4
Nucleus pulposus	1	0.49
Annulus fibrosus	4.2	0.45
Anterior longitudinal ligament (ALL)	7.8	0.3
Posterior longitudinal ligament (PLL)	10	0.3
Ligament flava (LF)	15	0.3
Supraspinous ligament (SSL)	8	0.3
Interspinous ligament (ISL)	10	0.3
Intertransverse ligament (ITL)	10	0.3
Capsular ligament (CL)	7.5	0.3
Aluminum alloy	110,000	0.28

### FE modelling of the surgical procedures

The internal fixation assembly and decompression model was constructed using UG software. The models were divided into four groups: M1, the intact; M2, unilateral decompression; M3, internal fixation alone; and M4, unilateral decompression combined with internal fixation (Fig. 1b). The ISDF device we used was BacFuse (RTI Surgical, Inc., Florida, USA). The geometry and material properties of the ISDF device provided by the instruction of this product. The material of the BacFuse was an aluminum alloy (Ti6Al4V). The size of the ISDF device was chosen based on the interspinous distance and 14 mm (distraction distance was 14 mm) was chosen to achieve suitable distraction in this study. The supraspinous ligament was retained and the interspinous ligament was removed when the ISDF device was implanted. The friction coefficients of various parts of the ISDF device were set to infinity. The spikes in the inner plate of the ISDF device were strongly fixed to the spinous process. This was a simulation of initial postoperative stage. Local laminectomy was performed to resect the lower part of the L4 lamina and the upper part of the L5 lamina at the right lamina of L4/5.

### Boundary and loading conditions

The inferior endplate of the L5 vertebra was fixed. A 400 N follower load was applied to the center of superior endplate of the L3 vertebra. Flexion, extension, left and right lateral bending and left and right rotation were performed at a moment of 7.5Nm [13, 16]. The range of motion (ROM) of the implanted and upper adjacent segment, IVDs stress, facet joint stress, interspinous process stress, and the internal fixation device stress were recorded.

## Results

### Model validation

**Fig. 2:**
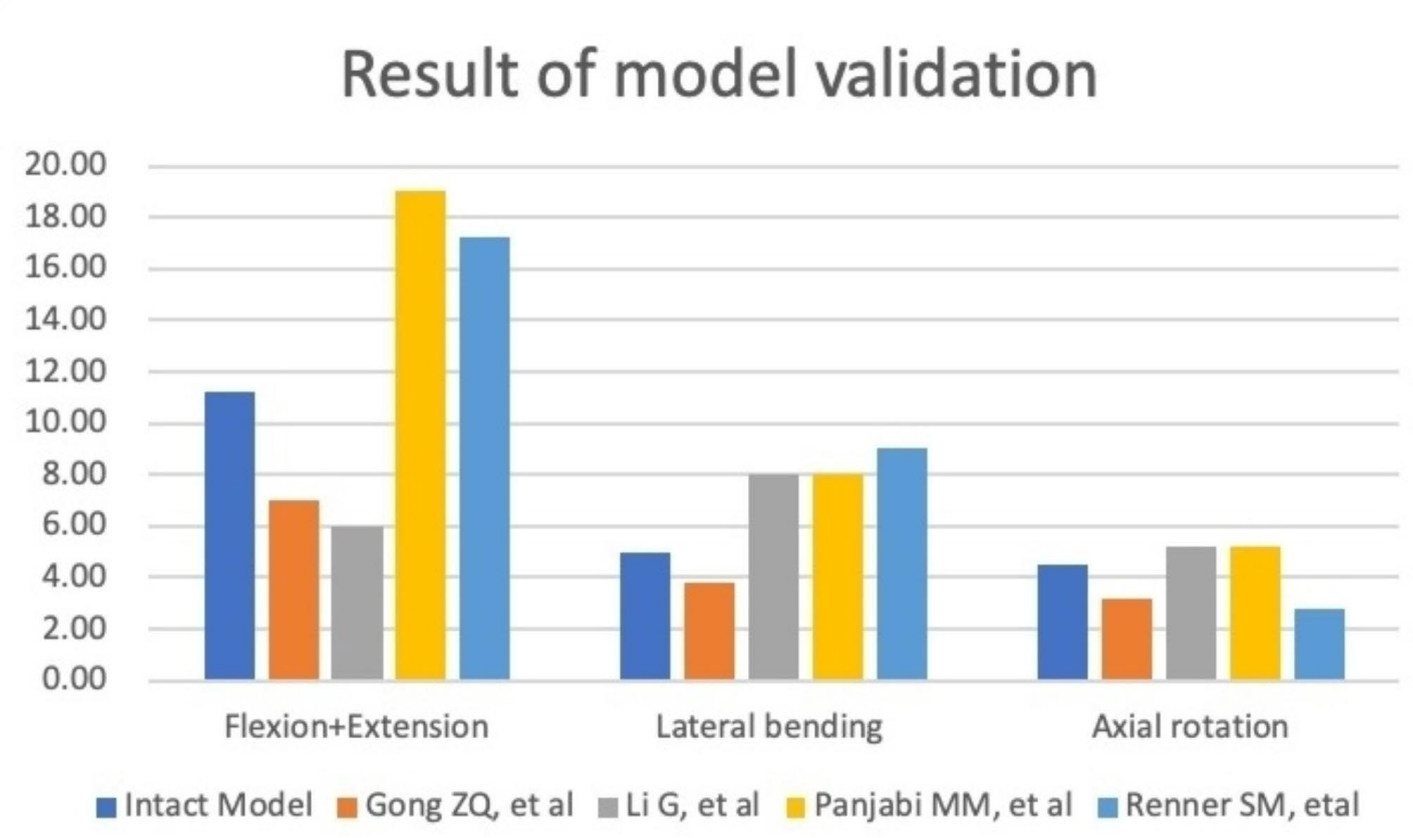
Comparison of intervertebral ROM between current intact model and previous literature. ROM, range of motion

FE models of L3-L5 were built and compared with the published literature on the ROM [17–20] (Fig. 2). The ROM of L3 to L5 were consistent with those previously published and were therefore used for further modelling and analysis.

### ROM of the implanted segment and upper adjacent segment

**Fig. 3:**
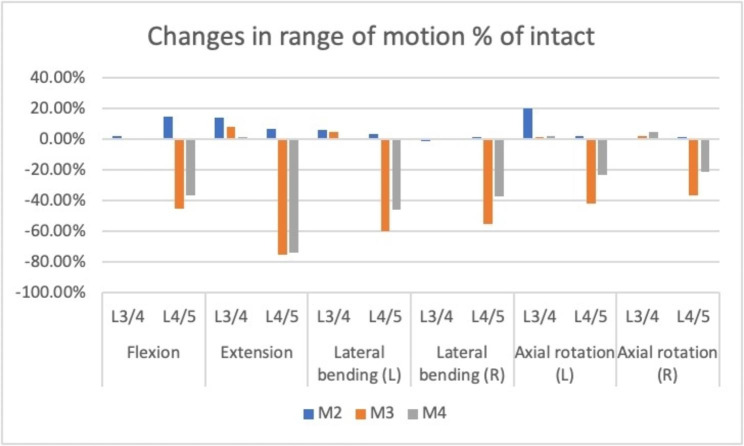
Changes in intervertebral ROM comparing the three intervention models to the intact model at the surgical and proximal level in the three models. ROM, range of motion

The changes in the intervertebral ROM compared with the intact model at the surgical and proximal levels in the three models are presented in Fig. 3.

In flexion, the ROM of L4/5 in M2 increased by 14.5% compared with that in M1. Compared with M1, the ROM was decreased in the M3 (-45.27%) and M4 groups (-36.39%) at L4/5. Compared with M1, the ROM of L3/4 was increased in the M2 (+ 2.03%), M3 (+ 0.81%), and M4 (+ 0.41%) groups. In extension, the ROM of L4/5 in M2 increased by 7.14% compared with that in M1. Compared with M1, extension was restricted in the M3 (-75.32%) and M4 (-74.03%) groups at L4/5. In the upper adjacent segment L3/4, the ROM of extension increased in the M2 (+ 14.04%), M3 (+ 7.89%), and M4 (+ 1.75%) groups.

In the left lateral bending, the ROM of L4/5 increased by 3.46% in M2, and decreased by 59.65% in M3 and 45.53% in M4. The ROM at L3/4 increased by 5.96% in M2, 5.11% in M3, and 0.15% in M4. In right lateral bending, the ROM of L4/5 increased by 1.78% in M2 compared with that in the intact group, and decreased by 54.90% in M3 and 37.39% in M4. Compared with M1, the ROM of L3/4 decreased by 1.15% in M2 and increased by 1.15% in M3 and M4.

In left rotation, the ROM of L4/5 in M2 increased by 1.92%, and decreased by 41.92% in M3 and 23.08% in M4 compared to M1. Compared with M1, the ROM of L3/4 increased by 20.18% in M2, 1.83% in M3, and 2.29% in M4. In right rotation, the ROM of L4/5 in M2 increased by 1.52% compared with that in M1, and decreased by 36.5% in M3 and 21.29% in M4. Compared with M1, the ROM of L3/4 increased by 2.4% in M3 and 4.81% in M4, with no significant change in M2.

### IVD stress in the implanted segment and upper adjacent segments

**Fig. 4:**
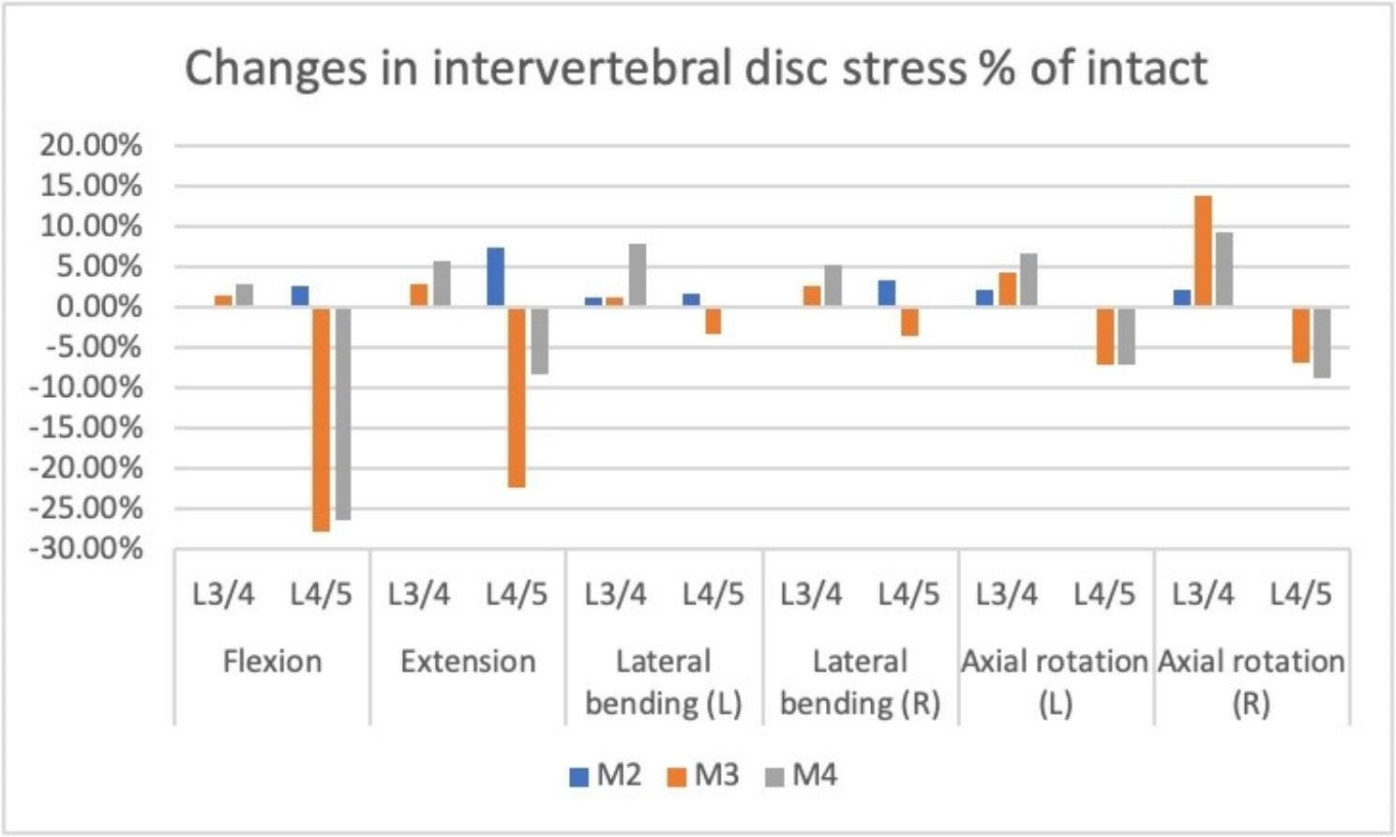
Changes in intervertebral stress comparing the three intervention models to the intact model at the surgical and proximal levels

**Fig. 5:**
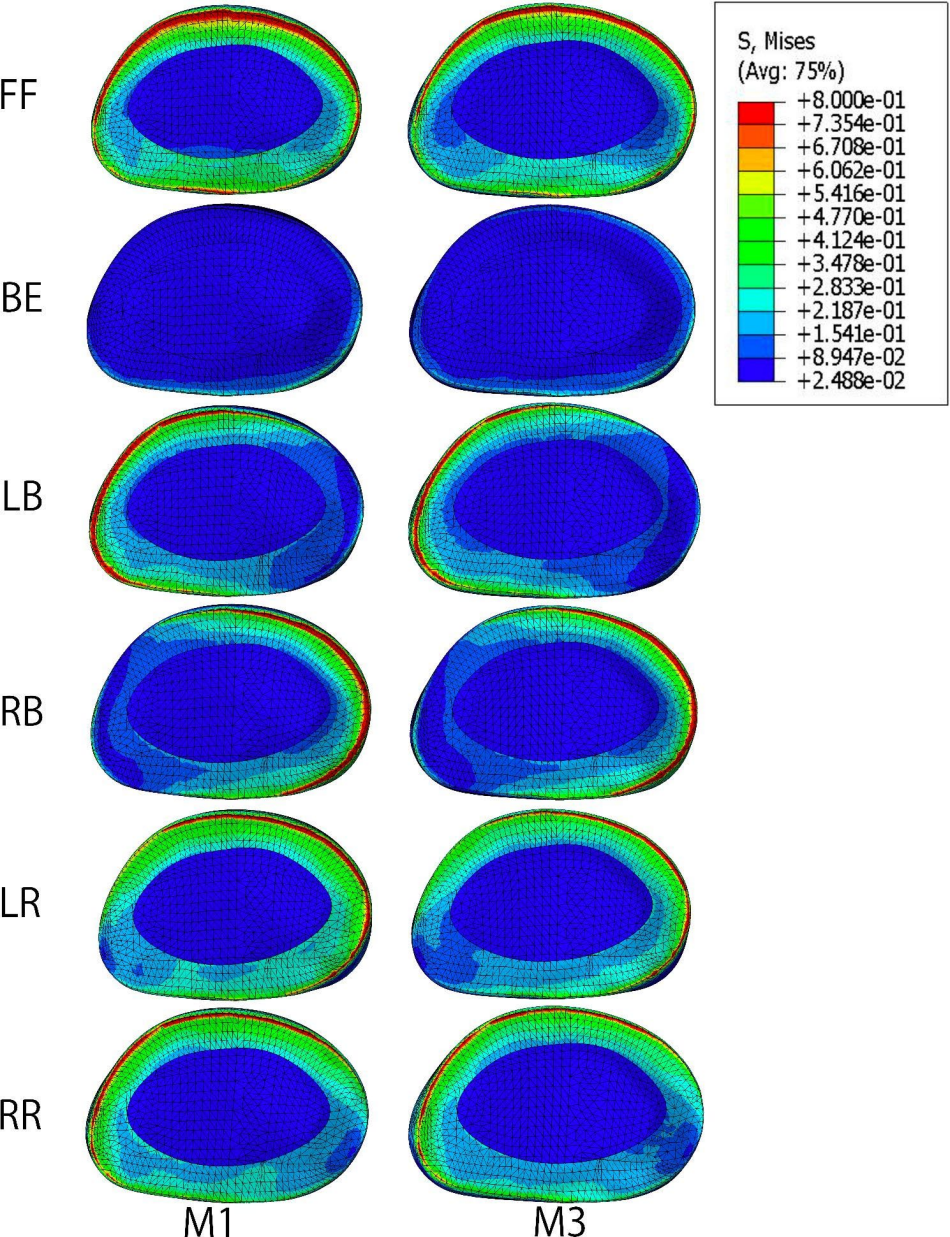
Intervertebral disc stress comparison between the intact model and the internal fixation alone model in different motions. FF, front flexion; BE, back extension; LB, left bending; RB, right bending; LR, left rotation; RR, right rotation

The maximum Von Mises stresses of the IVDs are shown in Fig. 4. The IVD in extension motion presented the lowest stress among all motion models. Compared with M1, the IVD stress in M3 decreased by 28%, 22%, 3%, 7% during flexion, extension, bending and rotation, respectively. The IVD stress cloud diagrams of L4/5 in M1 and M3 were presented in Fig. 5. The maximum stress distribution area (red region) in M3 decreased compared with M1. Compared with M2, the IVD stress in M4 decreased by 28%, 28%, 2%, 3%, 7%, and 9% during flexion, extension, left lateral bending, right lateral bending, left rotation, and right rotation, respectively.

Compared with M1, the upper adjacent IVD stress in M3 increased by 1.5%, 2.9%, 1.3%, 2.7%, 4.4%, and 14% during flexion, extension, left bending, right bending, left rotation, and right rotation, respectively. Compared with M2, the upper adjacent IVD stress in M4 increased by 3%, 5.7%, 6.6%, 5.4%, 9%, 11.9% during flexion, extension, left lateral bending, right lateral bending, left rotation and right rotation, respectively.

### Facet joint stress in the implanted segment and upper adjacent segments

**Fig. 6:**
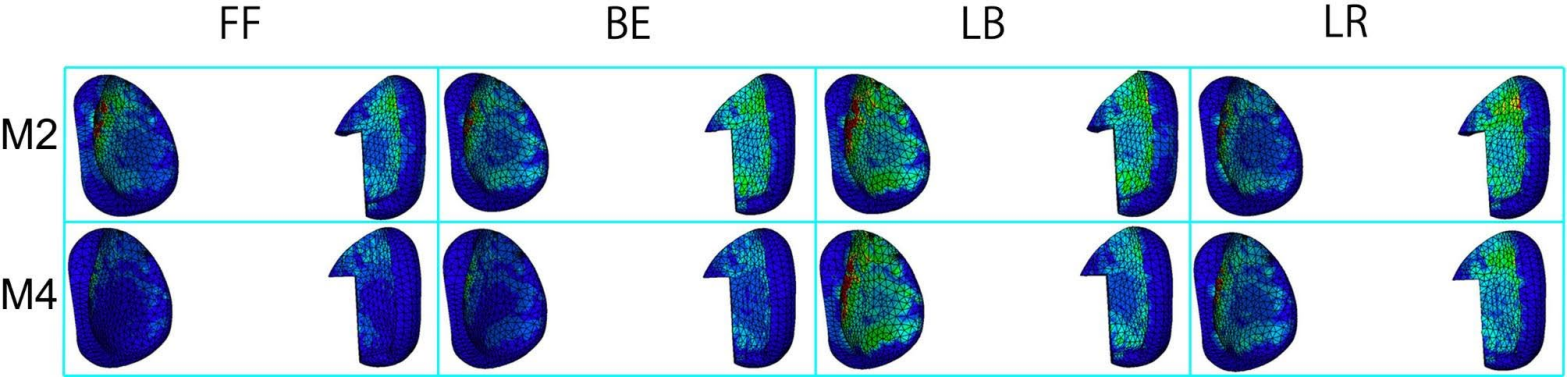
Facet joint stress distribution at L4/5 segment in M2 and M4. FF, front flexion; BE, back extension; LB, left bending; LR, left rotation

In the M1 and M2 models, the lateral bending motion increased the facet joint stress on the bending side, whereas the rotation motions did not cause a significant difference between the bilateral facet joints. The facet joint stresses in M2 did not increase after local decompression compared with M1. The facet joint stresses in M3 decreased at L4/5 during flexion, extension, lateral bending and rotation motions compared with those in M1. The facet joint stresses in M4 were significantly decreased in the L4/5 segments during flexion, extension, lateral bending and rotation motions compared with those in M2 (Fig. 6).

### Spinous process stress and the internal fixation device stress

**Fig. 7:**
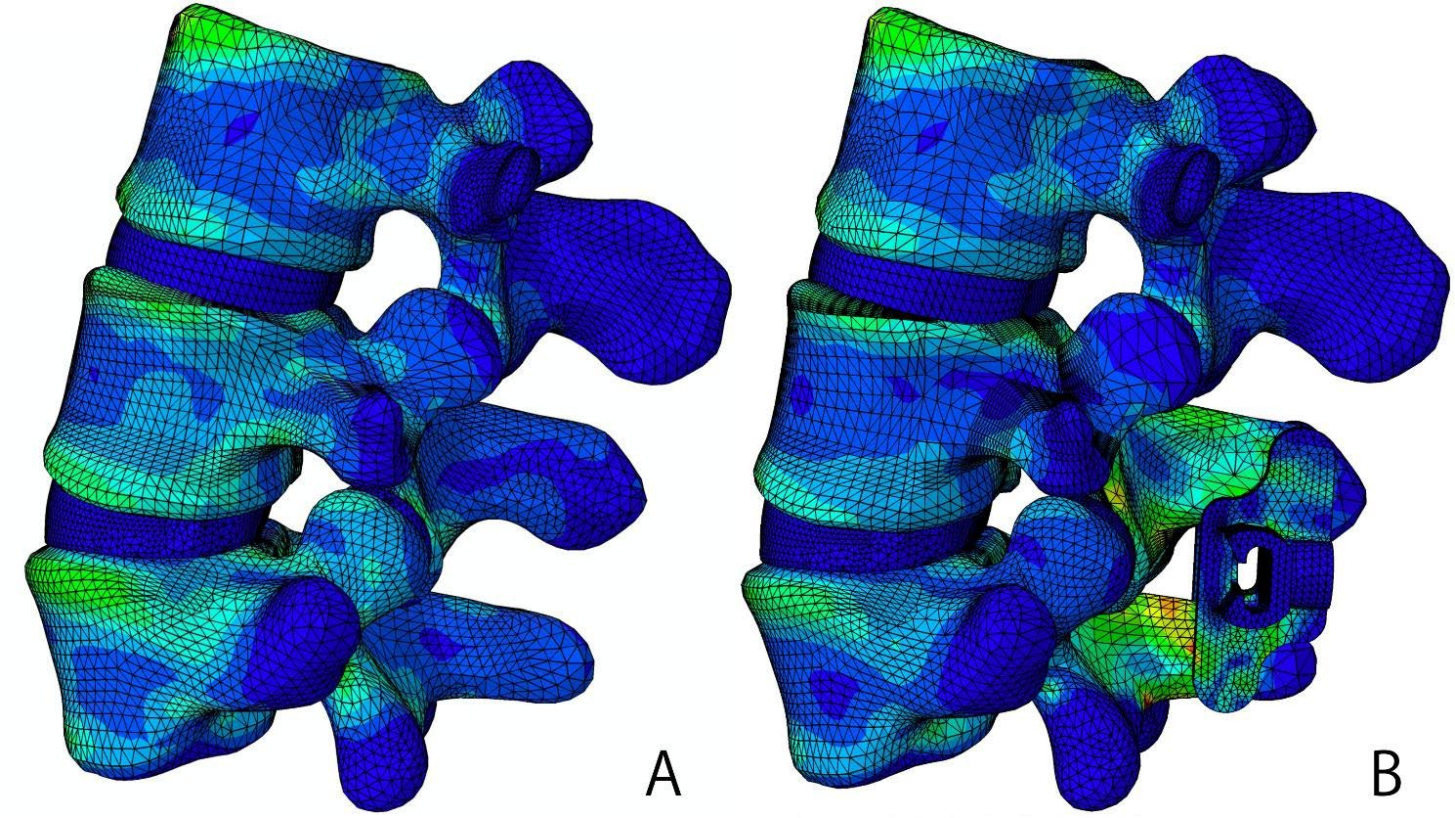
Lumbar vertebral stress distribution in flexion motion

After implanting the BacFuse, the spinous process was loaded with a certain amount of stress in all motion modes. The spinous process stress at L5 was higher than at L4 for all motion types. The vertebral body stress distribution during flexion motion is presented in Fig. 7.

**Fig. 8:**
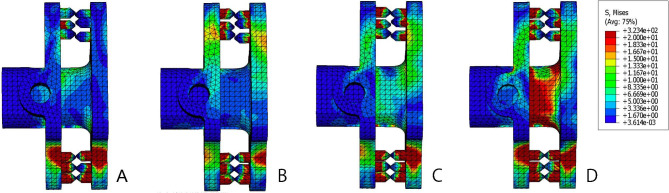
Internal fixation stress distributions in different motion directions. (**A**) Flexion. (**B**) Extension. (**C**) Lateral bending. (**D**) Rotation

The internal fixation stress distributions in different motion directions are presented in Fig. 8. The stress distribution of the internal fixation device is mainly concentrated on the spikes on the lateral plate, where the device is closely linked to the spinous process. Moreover, the stress on the inferior spikes was always higher than that on the superior spikes. The spacer of the device loads some of the stress. The spacer loaded the largest stress in the rotational motion; the next largest stress was in the lateral bending motion, and the smallest stress was in the flexion-extension motion.

## Discussion

Traditional IPD devices mainly aim to treat mild to moderate LSS by distracting the interspinous space and enlarging the spinal canal area to relieve the nerve root compression. IPD has shown good clinical efficacy in the early stages of LSS. However, the higher recurrence and revision rates during long-term follow-up have limited its applications [21, 22]. The main reasons for this are that indirect decompression alone does not guarantee clinical efficacy [4], and internal fixation device fatigue and bony erosion can result in symptom recurrence [3]. The ISDF device can be used alongside local laminectomy to increase stability. An interspinous bone graft can achieve interspinous local fusion to decrease the risk of internal fixation failure [5, 9]. In our previous study with 5 years follow-up, the satisfaction following ISDF reached 88.71%, being highest for lateral LSS, with good efficacy [23].

In this FE analysis study, we found that implantation of the ISDF device decreased the intervertebral ROM in flexion-extension, lateral bending, and rotational motions. The ROM in the internal fixation group decreased by 45.27%, 75.32%, 59.65%, 54.90%, 41.92% and 36.5% during flexion, extension, left lateral bending, right lateral bending, left rotation, and right rotation, respectively. This result is consistent with those of a previous study, in which Liu et al. found that the ROM decreased by 40.10%, 74.23%, 30.92%, and 24.39% during flexion, extension, lateral bending, and rotation, respectively, after implantation of the BacFuse. Similar to traditional IPD devices, BacFuse decreases the ROM in extension through the distraction. Previous studies have reported that X-stop and Coflex devices provide flexion-extension stability but have little effect on lateral bending and rotation [24, 25]. BacFuse restricts lateral bending and rotation through the strong clamping and locking mechanism of the lateral plates and spikes. This provides additional stability. Therefore, BacFuse is superior to traditional IPD devices in this respect.

Local laminectomy may influence the ROM of the decompressed segment. Wilke et al. reported that the ROM in lateral bending increased by 8% and rotation increased by 18% after laminectomy in a cadaveric biomechanical study [24]. In our study, the ROM of the local laminectomy segment increased by 14.50%, 7.14%, 3.46%, 1.78%,1.92%, and 1.52% during flexion, extension, left lateral bending, right lateral bending, left rotation, and right rotation, respectively. These results indicate that unilateral local laminectomy decreased flexion-extension stabilization. After implantation of the BacFuse, the ROM significantly decreased by 36.39%, 74.03%,45.53%, 37.39%, 23.08%, and 21.29% during flexion, extension, left lateral bending, right lateral bending, left rotation, and right rotation, respectively. After implanting traditional IPD devices (Coflex, Wallis, DIAM, and X-Stop), the ROM decreased by 50% during extension with no change in other motion directions [26]. Therefore, BacFuse can be used in combination with local laminectomy to achieve decompression and stability.

The ISDF can reduce the maximum stress of the IVD, especially during flexion, extension, and rotation motions. During flexion, the maximum IVD stress dropped by 27.78%. In extension, the maximum IVD stress dropped by 22.22%. During axial rotation, the maximum IVD stress decreased by 7.14%. Cheng-Chan et al. reported that IVD stresses decreased after implantation of the Coflex rivet in all motion models. The rivet connects the lateral wings and the bony spinous process providing greater stability than the traditional Coflex [27]. Similar to the Coflex rivet device, the ISDF device anchors the spinous process with the spikes in the lateral wings providing more stability. The more stability the device provides, the less stress IVD has to bear [28]. A reduction in IVD stress delays degeneration process [29]. It is also important to retain a certain of motion to maintain the disc viability [28].

From the stress cloud diagram, we found that the stress on annulus fibrosus was significantly higher than that on the nucleus pulposus. IVD stress was minimal during extension. The stress on the bending side was higher than that on the non-bending side during lateral bending. Rotational motion caused an increase in contralateral annulus fibrosus stress. A previous study reported that left lateral bending coupled with right rotation [30], which is consistent with the finding of our study. The IVD stress during left lateral bending was in the same area as that during right axial rotation.

The BacFuse reduced facet joint stress in the motions of flexion, extension, lateral bending and axial rotation. As a fulcrum, the ISDF device redirects force from the facet joint to the interspinous process. Lazaro et al. reported that the IPD reduced the mean facet load by 30% during flexion and 69% during extension in a nondestructive cadaveric flexibility testing [31].

After implantation of the ISDF device, the spinous process loads a certain amount of stress in any motion models. The spinous process bears not only a stretching force but also a compression force. Therefore, there is a risk of spinous process fractures. Different types of IPD devices have different effects on the spinous processes. Liu et al. reported that the maximum spinous stress occurred during extension. With the X-Stop, the L4 spinous process loaded the maximum contact forces, whereas with the Coflex, the L5 spinous process loaded the maximum contact forces [12]. In our study, the L5 spinous process bore more stress than the L4 spinous process in all motions. This may be due to the design of the BacFuse. The decompression of BacFuse mainly relies on laminectomy, rather than on the distraction of the interspinous space, in contract to X-Stop. The highest stress on the spinous process was at the site of linkage with the spikers of BacFuse. The spikes in BacFuse, especially the inferior spikes, also showed high stress. The device closely links to the spinous processes and mainly relies on the locking mechanism of the bilateral plate and the gripping of the bilateral spikes. Due to the shorter moments and higher young modulus of the spikes, higher stresses are more likely to cause spinous process erosion or fracture other than metal fatigue fracture in the long run. In contrast to BacFuse, the stress of traditional IPD devices is mainly concentrated in the spacer. In the BacFuse, only rotational motion showed maximum stress in the spacer.

After implantation of the BacFuse, the proximal segment ROM and IVD stress increased. This is consistent with the results obtained for other IPD devices. Compared with dynamic IPD devices, static IPD devices have a greater influence on adjacent segments [27]. However, BacFuse alleviated hypermobility and overload at upper adjacent levels compared to traditional fusion surgery [32, 33]. In our study, the BacFuse had a certain of influence on the upper adjacent segment degeneration.

The current FE study has several limitations. First, the model was derived from a lumbar disc herniation patient with slight lumbar degeneration. Most patients with lumbar degenerative diseases are elderly and may also have osteophytes, osteoporosis, and spinal deformities. This model was employed to estimate the general biomechanical characteristics of BacFuse using a straightforward and efficient approach. Second, the link between the spinous process and the spikes of the device was set in close contact during the experiment. In practice, they do not have a close fixation and there is a risk of displacement. Moreover, we did not implant a larger device to deliberately distract the interspinous space based on the idea of ISDF. FE experiments on devices of different sizes will be performed in the future. Last, we used the anatomy of a single subject, so generalizability is limited.

## Conclusion

BacFuse exhibited a reduction in intervertebral ROM, as well as decreased stress on the intervertebral disc and facet joint, while also demonstrating a discernible impact on the upper adjacent segment.

## Data Availability

The datasets used during the current study are available from the corresponding author on reasonable request. Readers can access the data and material supporting the conclusions of the study by contacting Mengmeng Chen at chenmengmeng112233@163.com.
